# Protein arginine methyltransferase 1 is required for maintenance of normal adult hematopoiesis

**DOI:** 10.7150/ijbs.38859

**Published:** 2019-10-23

**Authors:** Lei Zhu, Xin He, Haojie Dong, Jie Sun, Hanying Wang, Yinghui Zhu, Feiteng Huang, Jingying Zou, Zexin Chen, Xiaoying Zhao, Ling Li

**Affiliations:** 1 Department of clinical laboratory, The Second Affiliated Hospital, Zhejiang University School of Medicine, No. 88 Jiefang Road, Hangzhou, 310009, Zhejiang, China.; 2 Department of Hematological Malignancies Translational Science, Gehr Family Center for Leukemia Research, Hematologic Malignancies and Stem Cell Transplantation Institute, Beckman Research Institute, City of Hope Medical Center, Duarte, CA 91010.; 3 Department of Science and Development, The Second Affiliated Hospital, Zhejiang University School of Medicine, No. 88 Jiefang Road, Hangzhou, 310009, Zhejiang, China.; 4 Department of Hematology, The Second Affiliated Hospital, Zhejiang University School of Medicine, No. 88 Jiefang Road, Hangzhou, 310009, Zhejiang, China.

**Keywords:** PTMT1, HSPCs, normal hematopoiesis

## Abstract

Protein arginine methyltransferase 1 (PRMT1) is the predominant asymmetric (type I) methyltransferase in mammalian cells. Mounting evidence suggested that PRMT1 is essential to embryonic development and tumor pathogenesis, but its role in normal adult hematopoiesis is less studied. We used a *Prmt1* conditional knockout (KO) mouse model to identify the role of PRMT1 in normal adult hematopoiesis. The results indicated that deletion of PRMT1 results in anemia and leukopenia, reducing terminal erythroid and lymphocyte differentiation. Additionally, we found a significant decrease of megakaryocyte progenitors (MkPs) compared with similarly treated littermate control mice. The frequency of short-term hematopoietic stem cells (ST-HSCs) and granulocyte-macrophage progenitors (GMPs) populations were significantly lower in PRMT1^f/f^/Mx1-CRE bone marrow (BM) compared with littermate control mice. Importantly, in-vitro replating assays and BM transplantation results revealed that PRMT1 KO results in reduced hematopoietic stem and progenitor cells (HSPCs) self-renewal capacity. Thus, we conclude that PRMT1 is required for hematopoietic differentiation and the competitive fitness of HSPCs, and we believed that PRMT1 serves as a key epigenetic regulator of normal hematopoiesis that occurs throughout life.

## Introduction

Protein arginine methylation is one of the most common posttranslational modifications that affects the function of many proteins, including both histone and non-histone proteins. A family of enzymes, namely, protein arginine methyltransferases (PRMTs), catalyze the formation of mono- or dimethyl arginine on a variety of protein substrates 1, thereby regulating many cellular processes including RNA processing, gene transcription, DNA damage repair, signal transduction and chromosome translocation 2. PRMT1 is the predominant asymmetric (type I) methyltransferase in mammalian cells, responsible for over 85% of arginine methylation activity. Mounting evidence in cancers, including our own results in leukemia, indicates that aberrantly overexpressed PRMT1 could serve as a potential therapeutic target 3-10. Homozygous PRMT1 deletion is embryonically lethal 11, 12, but its role in normal adult hematopoiesis is less studied. Others have reported that PRMT1 function is involved in normal B cell differentiation 13. Some reported that PRMT1, functioning through p38a, serves as a novel molecular switch in lineage-specific differentiation toward megakaryocytes or erythrocytes 14, 15. While the detailed function of PRMT1 in adult hematopoietic stem and progenitor cells (HSPCs) self-renewal and differentiation is still largely unknown. In this study, we identified the role of PRMT1 in normal adult hematopoiesis using a *Prmt1* conditional knockout (KO) mouse model (PRMT1^f/f^/Mx1-Cre). Overall, PRMT1 deletion in adult mice leads to anemia and leukopenia, thereby disrupting normal hematopoiesis. Although PRMT1 KO barely affected the primary mouse LSK frequency, BM transplantation studies revealed that PRMT1 KO results in reduced competitive fitness of HPSC.

## Materials and Methods

### Mice

*Prmt1* conditional KO mice (PRMT1^f/f^/Mx1-Cre) were generated by crossing PRMT1^f/f^ mice 16 with Mx1-Cre mice. To induce *Prmt1* deletion, 6- to 8-week-old PRMT1^f/f^ /Mx1-Cre mice were injected intraperitoneally with 14 mg/kg per dose of polyinosinic-polycytidylic acid (pIpC; InvivoGen) every other day for 7 doses. Similarly treated littermates lacking Mx1-cre alleles were used as control. All mice were maintained and all procedures were performed in accordance with federal and state government guidelines and established institutional guidelines and protocols approved by the Institutional Animal Care and Use Committee at City of Hope.

### Flow Cytometry

Femurs and tibias were crushed with a mortar and pestle to collect BM cells. Spleens were crushed with the end of a plunger. Cells were resuspended in 5 mL phosphate buffered saline (PBS) plus 0.5% bovine serum albumin (BSA), then filtered through a 70- μm filter (BD Biosciences) following by red blood cell lysis. Antibodies were used for flow-cytometry analyses as follows: CD117 (ckit, clone ACK2, eBioscience), Ly-6A/E (Sca-1, clone D7,BioLegend), CD150 (SLAM, clone mShad150, BioLegend,), CD48 (clone HM48.1, BioLegend), CD34 (clone MEC14.7, eBioscience), CD16/CD32 (clone 93, eBioscience), CD127 (IL7R, clone A7R34,eBioscience), CD135 (Flk-2, Flt-3, Ly-72, clone A2F10, eBioscience),CD45.1 (clone A20, BioLegend), CD45.2 (clone 104,BioLegend), CD11b (clone M1/70, eBioscience), Ter119 (cloneTER-119, BioLegend), B220(clone RA3-6B2, eBioscience ), CD3 (clone 17A2, eBioscience) and Ly-6G/Ly-6C (Gr1, cloneRB6-8C5, BioLegend). The lineage antibody cocktail included the following biotin-conjugated anti-mouse antibodies: CD19 (clone eBio1D3),NK-1.1 (clone PK136), B220 (clone RA3-6B2), IgM (clone II/ 41), CD3 (clone 17A2), CD4 (clone GK1.5), CD8 (clone s3-6.7), Gr1 (clone RB6-8C5), CD127 (clone A7R34) at 1 µg/mL,CD11b (clone M1/70),CD11c(clone N418) at 2 µg/mL, and Ter119 (clone Ter119, from BioLegend) at 3 µg/mL. Secondary antibody for the analysis was streptavidin-FITC (BioLegend). Flow cytometry was performed using a 5-laser, 15-detector Foressa X20 (BD Biosciences). Acquired data were analyzed by Flowjo software (TriSTAR).

Phenotypic populations were defined as LSK(Lin^-^/ckit^+^/Sca1^+^), long-term HSCs (LT-HSCs) (Lin^-^/ckit^hi^/Sca1^+^/Flt3^-^/CD150^+^/CD48^-^), short-term HSCs (ST-HSCs) (Lin^-^ckit^hi^/Sca1^+^/Flt3^-^/CD150^-^/CD48^-^), multipotent progenitors (MPPs) (Lin^-^/ckit^hi^/Sca1^+^/CD150^+/-^/CD48^+^), lymphoid-primed multipotent progenitors (LMPPs)(Lin^-^/ckit^hi^/Sca1^+^/Flt3^+^/CD150^+/-^/CD48^+^), common lymphoid progenitors (CLPs)(Lin^-^/IL7R^+^/ckit^lo^/Sca1^lo^), myeloid progenitors (MPs) (Lin^-^/ckit^+^/Sca1^-^), common myeloid progenytors (CMPs) (Lin^-^/ckit^+^/Sca1^-^/CD34^+^/FcgR^lo^), granulocyte-macrophage progenitors (GMPs) (Lin^-^/ckit^+^/Sca1^-^/CD34^+^/FcgR^hi^), and megakaryocyte-erythroid progenitors (MEPs) (Lin^-^/ckit^+^/Sca1^-^/CD34^-^/FcgR^lo^).

### Colony-forming cell assays

Myeloid/erythroid colony-forming units (CFUs; CFU-GEMMs, CFU-GMs, and BFU-Es) were enumerated using MethoCult containing stem-cell factor (SCF), IL-3, IL-6, and erythropoietin (EPO) (M3434; Stem Cell Technologies). Briefly, 1
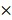
10^5^ cells per mL per dish were seeded in MethoCult and were counted at day 7 according to the manufacturer's protocol. For replating assays, cells from each plate were harvested and replated at 1 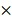 10^5^ cells per mL per dish after 7 days CFC culture.

### Transplantation Experiments

For competitive repopulation experiments, primary donor BM cells (1 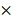 10^6^) isolated from PRMT1^f/f^/Mx1-Cre or PRMT1^f/f^ mice (which are CD45.2), mixed with equal numbers of CD45.1 competitor cells and injected via tail vein into lethally irradiated (11 Gy; 2 split doses) 6- to 8-week-old C57BL/6 congenic CD45.1^+^ recipient mice. After robust engraftment of donor cells around 4 weeks post-bone marrow transplant (BMT), mice were injected intraperitoneally 7 doses (14 mg/kg per dose) of pIpC. Donor chimerism in peripheral blood (PB) was evaluated over time till 12 weeks for BM engraftment analysis.

For noncompetitive BM primary transplantation, 2 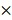 10^6^ primary donor BM cells isolated from PRMT1^f/f^/Mx1-Cre or PRMT1^f/f^ mice (both are CD45.2^+^) were injected via tail vein into lethally irradiated congenic CD45.1^+^ recipient mice. Mice were euthanized for phenotypic analysis at indicated time points.

### Statistics

Statistical analyses were performed with Student's t test or ANOVA for normal distribution. Mann-Whitney U tests were performed when normal distribution was not satisfied. *P* value less than 0.05 was considered statistically significant.

## Results

### Generation of PRMT1 conditional knockout mice

**Figure 1:**
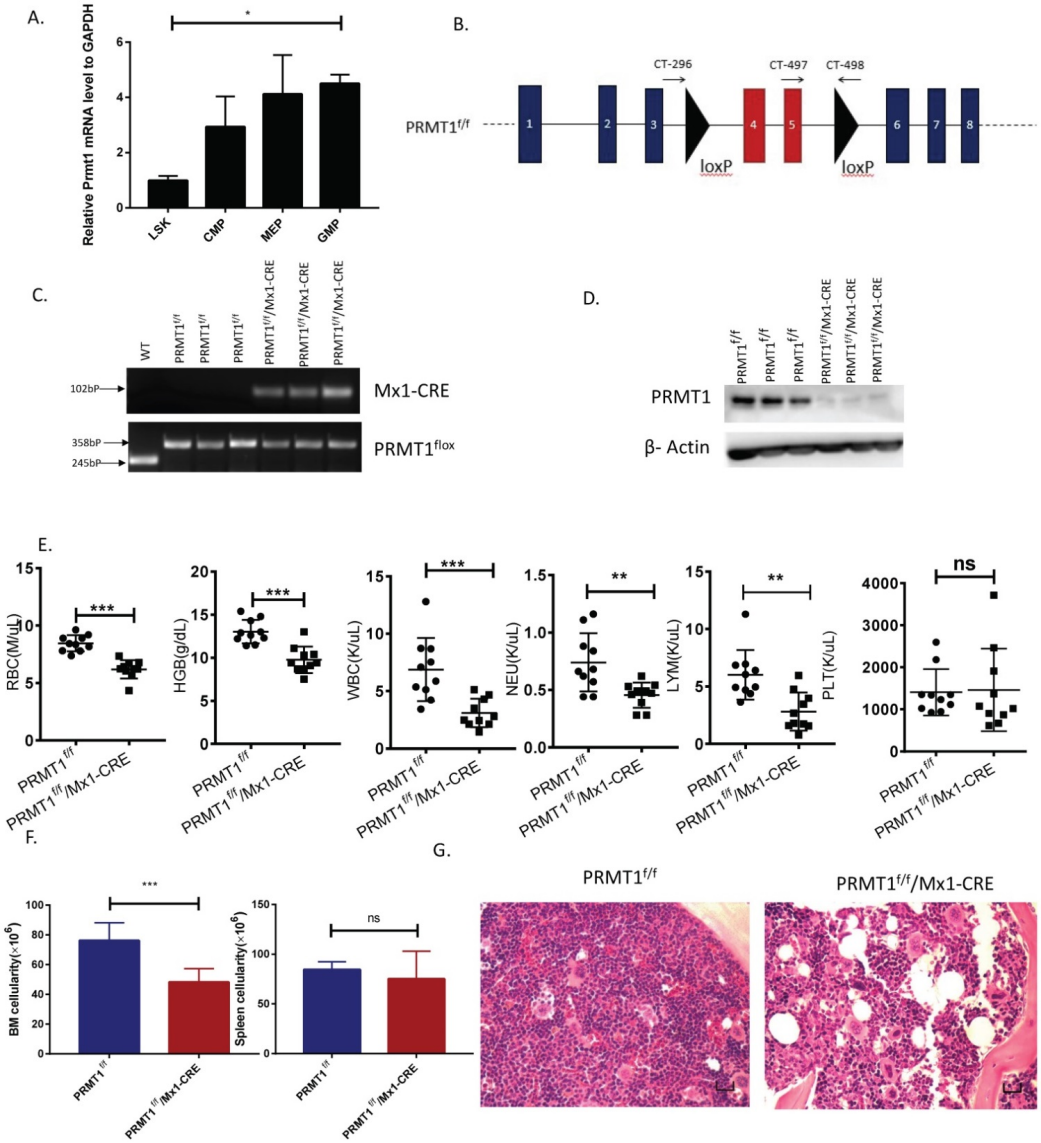
** Deletion of PRMT1 results in anemia and leukopenia. (**A) Relative* Prmt1* mRNA levels in indicated HSPCs populations, detected by quantitative reverse transcriptase polymerase chain reaction (qRT-PCR; *n*=3, each performed in duplicate).*p<0.05, p value was determined by one way ANOVA. (B) Generation of a PRMT1 flox allele in mice. Representation of the floxed exons are the blue boxes, and the line represents introns. The black triangles denote loxP sites, and the small arrows denote the primers used for PCR analysis. The PRMT1 conditional allele consists of exons 4 and 5, which encode part of the methyltransferase domain, flanked by loxP sites. (C) Genomic DNA was purified from tails for further PCR analysis. Tissue from 3 different PRMT1^f/f^ and PRMT1^f/f^/Mx1-CRE mice was used (*n*=3). Performed in duplicate from 3 independent experiments. (D) PRMT1 protein levels were determined by Western blot analysis using PRMT1^f/f^ or PRMT1^f/f^/Mx1-CRE mouse BM cells (*n*=3). Performed in duplicate from 3 independent experiments. (E) Complete blood count (CBC) analysis of peripheral blood cell RBC, Hb, WBC, neutrophil, lymphocyte and Plt counts at 12 weeks after pIpC injection are shown(*n*=10).**p<0.01,***p<0.001. p values were determined by Mann-Whitney U tests. (F) Number of total BM and spleen cells from PRMT1^f/f^ and PRMT1^f/f^/Mx1-CRE mice (*n*=10). ***p<0.001. p value was determined by Mann-Whitney U tests. (G) Representative H&E- staining image shows cross sections of femurs isolated from the PRMT1^f/f^ and PRMT1^f/f^/Mx1-CRE mice (scale bars, 100µm).

To determine PRMT1 function in normal hematopoiesis, we first measured *Prmt1* gene expression levels in multiple hematopoietic subpopulations from C57BL/6 mice (6- to 8-week-old). We sorted different subpopulations according to phenotypic markers and then *Prmt1* expression levels were determined by quantitative RT-qPCR. There is no statistical significant difference of *Prmt1* mRNA levels among CMP, MEP and GMP, and the level of *Prmt1* mRNA in the phenotypic LSK was lower relative to GMP (Fig. 1A).

To determine PRMT1 function in hematopoiesis, we have developed conditional *Prmt1* KO mouse (PRMT1^f/f^/Mx1-Cre) by crossing Mx1-Cre transgenic mouse with Prmt1-floxed (Prmt1^f^) mice as previously described 7. The PRMT1 conditional allele consists of exons 4 and 5, which encode part of the methyltransferase domain, flanked by loxP sites. Deletion of these exons creates a frameshift, resulting in a functionally null allele (Fig. 1B). We induced *Prmt1* deletion of adult hematopoietic cells by injection of 7 doses of pIpC (14 mg/kg every other day, injection, i.p.), and simultaneously, pIpC-treated littermates (Mx1-Cre-negative) were included as controls. PRMT1 flox alleles were confirmed by PCR analysis (Fig. 1C). PRMT1 deletion at protein levels was confirmed by Western blot analysis (Fig. 1D).

### Deletion of PRMT1 results in anemia and leukopenia

Overall, deletion of PRMT1 had a prominent effect on adult hematopoiesis in 6- to 8-week-old mice. Analysis of the peripheral blood of these mice revealed significant decreases in red blood cells (RBCs), hemoglobin (Hb) and white blood cells (WBCs) at 12 weeks post pIpC induction and the absolute number of neutrophils and lymphocytes were decreased (Fig. 1E). Notably, RBC and Hb counts were starting to decrease as early as 4 weeks after pIpC induction (Supplemental Fig. 1) compared with littermate controls. Importantly, BM cellularity of PRMT1-KO mice was decreased compared with littermate controls (Fig. 1F left, histologic examination was shown in Fig. 1G) but, PRMT1 deletion did not have obvious effects on spleen cellularity at 12 weeks post pIpC induction (Fig. 1F right). Moreover, the *Prmt1* heterozygous KO mice had normal peripheral blood counts and normal BM, spleen cellularity (data not shown). Intriguingly, approximately one out of eight of the PRMT1^f/f^/Mx1-CRE mice died within 5 months, whereas none of the PRMT1^f/f^ mice (*n*=6) died at the end point of our evaluation (Supplemental Fig. 2).

### Loss of PRMT1 affects terminal hematopoietic differentiation

**Figure 2:**
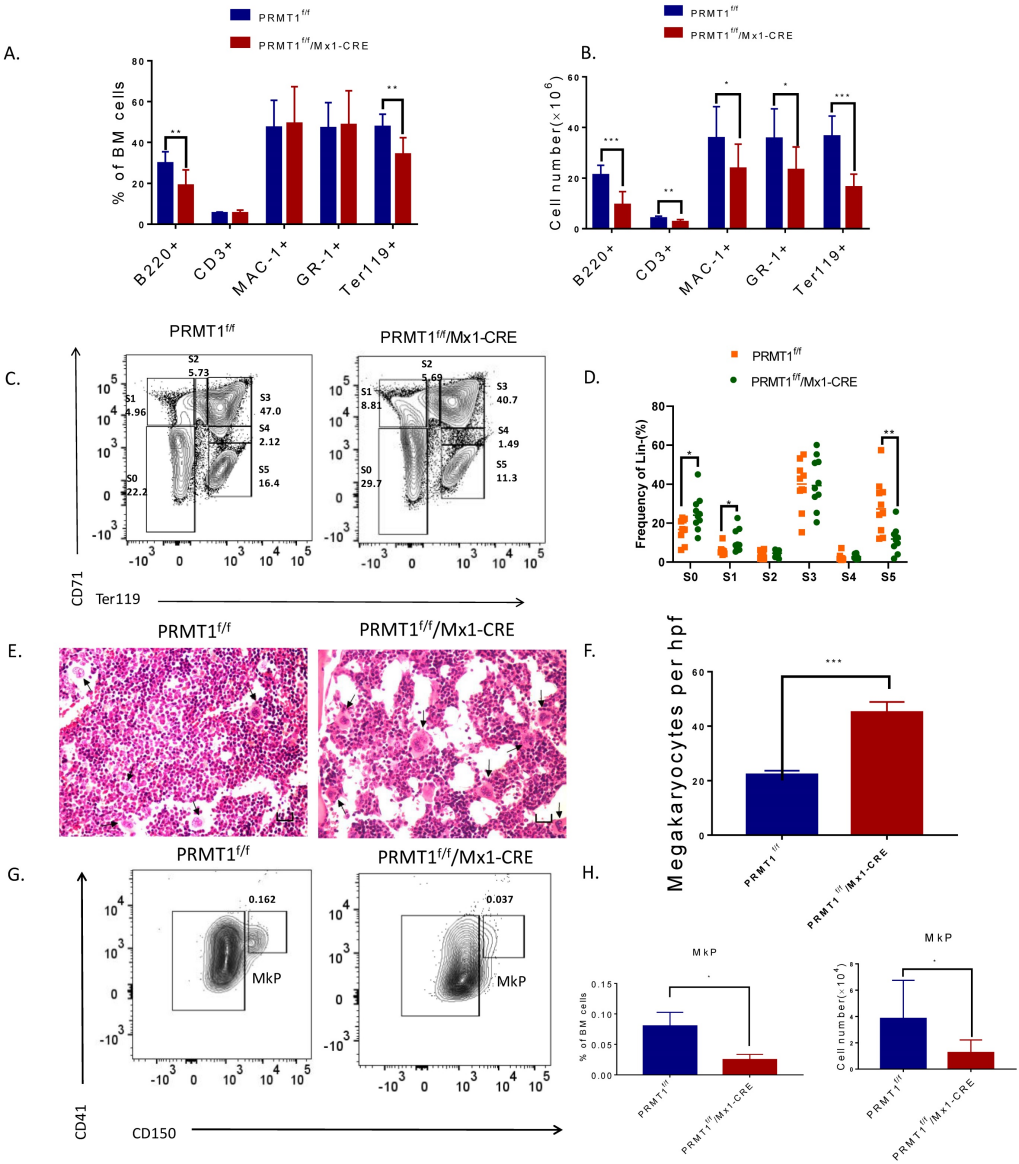
** Loss of PRMT1 affects terminal hematopoiesis differentiation. (**A) Frequency of CD3/B220, Mac1/Gr1 and Ter119 population in PRMT1^f/f^/Mx1-CRE and PRMT1^f/f^ mouse BM cells (*n*=10). **p<0.01. p values were determined by Mann-Whitney U tests. (B) Number of CD3/B220, Mac1/Gr1 and Ter119 population in PRMT1^f/f^/Mx1-CRE and PRMT1^f/f^ mouse BM cells (*n*=10). The absolute number of each subpopulation is calculated through multiply total BM cell number by BM percentage. *p<0.05, **p<0.01, ***p<0.001. p values were determined by Mann-Whitney U tests. (C) Representative FACS plots showing various erythroid progenitor subsets defined by the expression of Ter119 and CD71 in PRMT1^f/f^/Mx1-CRE and of PRMT1^f/f^ mouse BM analyzed by flow cytometry. (D) Representative FACS plots showing gating strategy and the frequency of each phenotypic erythroid subset (S_0_-S_5_) in Lin^-^ BM (*n*=10).*p<0.05, **p<0.01. p values were determined by Student's t test. (E) H&E staining of the femur section of PRMT1^f/f^/Mx1-CRE and of PRMT1^f/f^ mice, megakaryocytes are marked with arrows (scale bars, 100µm). (F) Number of megakaryocytes per high power field (hpf) in the BM evaluated in PRMT1^f/f^/Mx1-CRE and PRMT1^f/f^ mice (*n*=5). ***p<0.001. p value was determined by Student's t test. (G) Representative FACS plots showing gating strategy and frequency of MkP populations from PRMT1^f/f^/Mx1-CRE and of PRMT1^f/f^ mouse BM. (H) Frequency and numbers of MkP in total BM cells (*n*=5). *p<0.05. p value was determined by Student's t test.

To evaluate the effects of PRMT1 deletion on adult hematopoiesis, we first determined the frequency of erythroid (Ter119+), mature myeloid (Mac1+/Gr1+), B cells (B220) and T cells (CD3) in the BM of PRMT1-deleted mice and found that Ter119+ and B220+ subsets were significantly decreased compared with littermate controls, while the myeloid and T cell populations were relatively intact (Fig. 2A). Importantly, we also calculated the absolute numbers of erythroid, myeloid, T and B cells on the basis of their frequencies and BM cellularity and observed overall decreases in all lineage cells (Fig. 2B). The observation of B cell maturation defects is consistent with other reports using B cell specific PRMT1 KO mice 17-19.

Moreover, CD71 and Ter119 markers were used to define the maturation stages of erythroid progenitors (Fig. 2C). In PRMT1 KO mice, we observed significantly enriched primitive erythroid subsets including S0 (CD71^-^/Ter119^-^) as well as S1 (CD71^hi^/Ter119^-^) populations, accounting for a relative decrease of mature erythroid cells (S5, CD71^-^Ter119^hi^, Fig. 2D), thereby indicating erythroid maturation block.

Interestingly, our histology analysis of BM showed hypocellularity with megakaryocytosis. Clusters of megakaryocytes were commonly observed in BM of PRMT1 KO mice (Fig. 2E, F), indicating more differentiation to the lineage of megakaryocytes. Additionally, our analysis also showed a significant decrease of megakaryocyte progenitors (MkPs, defined as Lin^-^ckit^+^scal^-^CD41^+^CD150^+^) compared with similarly treated littermate control mice (Fig. 2G,H). These results are consistent with previous results 15, 20, confirming the important role of PRMT1 in antagonizing megakaryocyte differentiation.

### PRMT1 deletion result in adult mouse HSPCs reduction

**Figure 3:**
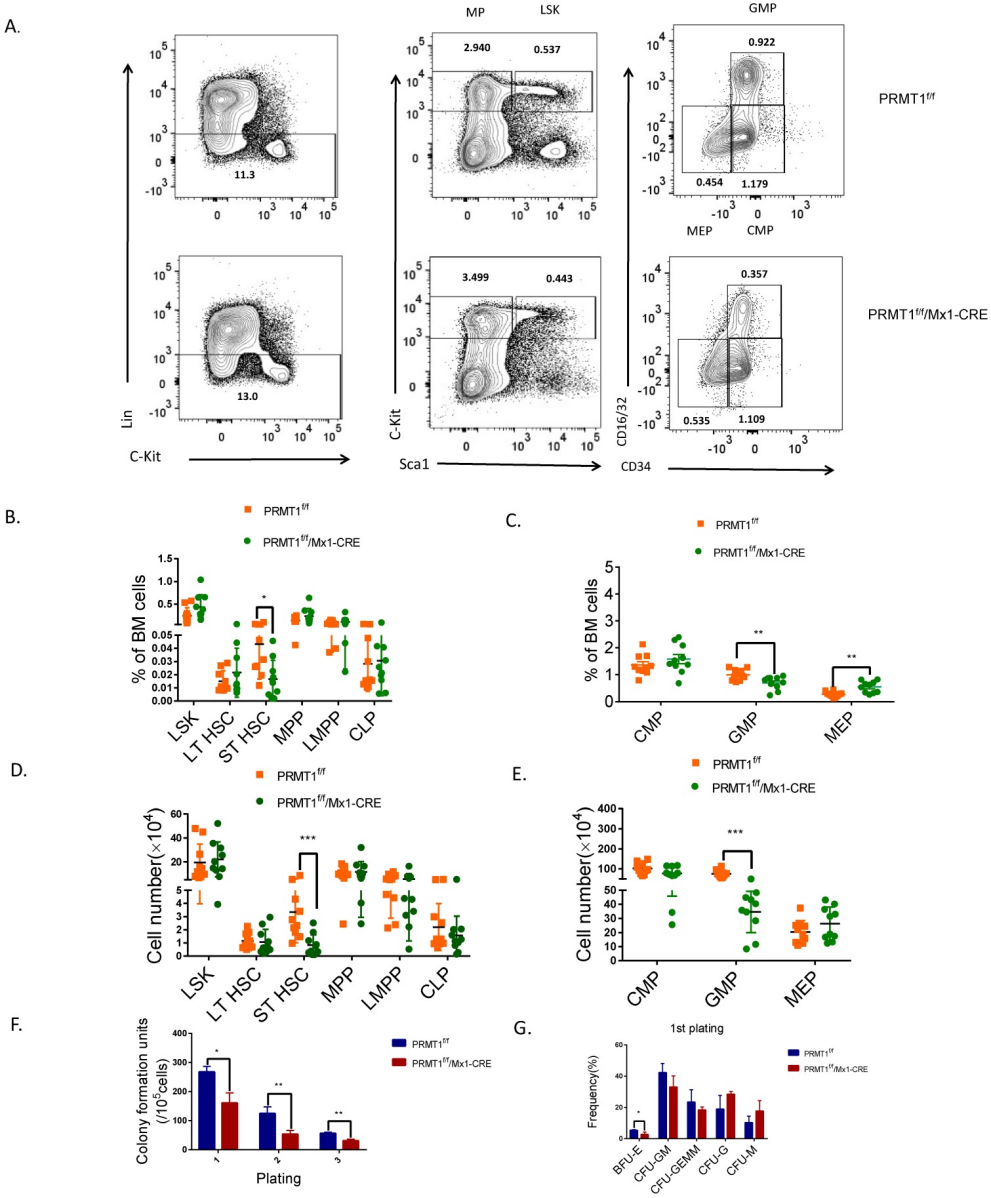
** PRMT1 deletion results in adult mouse HSPCs reduction. (**A) Representative FACS plots showing gating strategy and frequency of phenotypic populations including LSKs, MPs, CMPs, GMPs and MEPs from PRMT1^f/f^/Mx1-CRE and of PRMT1^f/f^ mouse BM. (B) Frequency of LSK, LT-HSC, ST-HSC, MPP, LMPP and CLP populations in PRMT1^f/f^/Mx1-CRE and of PRMT1^f/f^ mouse BM (*n*=10). *p<0.05. p value was determined by Student's t test. (C) Frequency of CMP, GMP and MEP subsets in PRMT1^f/f^/Mx1-CRE and of PRMT1^f/f^ mouse BM (*n*=10).**p<0.01. p value was determined by Student's t test. (D) Number of LSK, LT-HSC, ST-HSC, MPP, LMPP and CLP populations in PRMT1^f/f^/Mx1-CRE and of PRMT1^f/f^ mouse BM (*n*=10). **p<0.01. p value was determined by Mann-Whitney U tests. (E) Number of CMP, GMP and MEP subsets in PRMT1^f/f^/Mx1-CRE and of PRMT1^f/f^ mouse BM (*n*=10). ***p<0.001. p value was determined by Mann-Whitney U tests. (F) Colony-forming progenitor (CFU) assays were performed using PRMT1^f/f^/Mx1-CRE and PRMT1^f/f^ mouse bone marrow cells. CFU- number was shown. Cells were replated over 2 rounds of weekly successive replating (P2, P3). Performed in duplicate from 3 independent experiments.*p<0.05, **p<0.01. p values were determined by Student's test. (G) Percentage of BFU-E, CFU-GM, CFU-GEMM, CFU-G and CFU-M colonies at 1^st^ plating. *p<0.05. Performed in duplicate from 3 independent experiments. p value was determined by Student's test.

To explore the biology of PRMT1-deficient HSPC, we first analyzed the frequency and absolute cell number of BM HSPC populations from PRMT1-KO mice by flow cytometry. PRMT1 deletion did not affect the frequency of lineage-negative cells (that do not express Ter119, Mac1, Gr1, B220, CD3e, CD4, CD5, CD8 or CD127) (Fig. 3A). The frequencies of phenotypic progenitor subsets in BM, including LSK (Lin^-^/cKit^hi^/Sca1^+^),MPP and LMPP was not affected upon PRMT1 KO (Fig. 3B). Notably, the frequency of the ST-HSC population and GMPs was significantly lower in PRMT1^f/f^/Mx1-CRE BM compared with littermate control mice (Fig. 3B, C). We also noticed that the frequency of MEPs was higher in PRMT1 KO compared with littermate controls (Fig. 3C). Moreover, the absolute cell numbers of ST-HSCs and the GMPs subset was significantly decreased compared with littermate control mice (Fig. 3D, E).

We next evaluated the function of PRMT1 KO hematopoietic progenitor cells using an *in vitro* colony-forming cell (CFC) assay. After pIpC induction, total BM cells (1×10^5^ cells) from PRMT1^f/f^/Mx1-CRE or PRMT1^f/f^ mice were seeded in semisolid methylcellulose culture media supplemented with SCF, IL-3, IL-6, and EPO. PRMT1^f/f^/Mx1-CRE progenitors generated lower numbers of CFUs, also characterized with smaller size at first plating (Fig. 3F). We then performed serial replating every 7 days to assess their capacity to maintain progenitor activity over time. Significantly reduced numbers of CFUs were produced from PRMT1^f/f/^Mx1-CRE progenitor cells in secondary or tertiary replating (Fig. 3F). Differential counts of colony morphology revealed that PRMT1^f/f^/Mx1-CRE cells produced a reduced frequency of BFU-E compared with control cells, especially in the first plating (Fig. 3G).

### PRMT1 deficiency impairs competitive fitness

**Figure 4:**
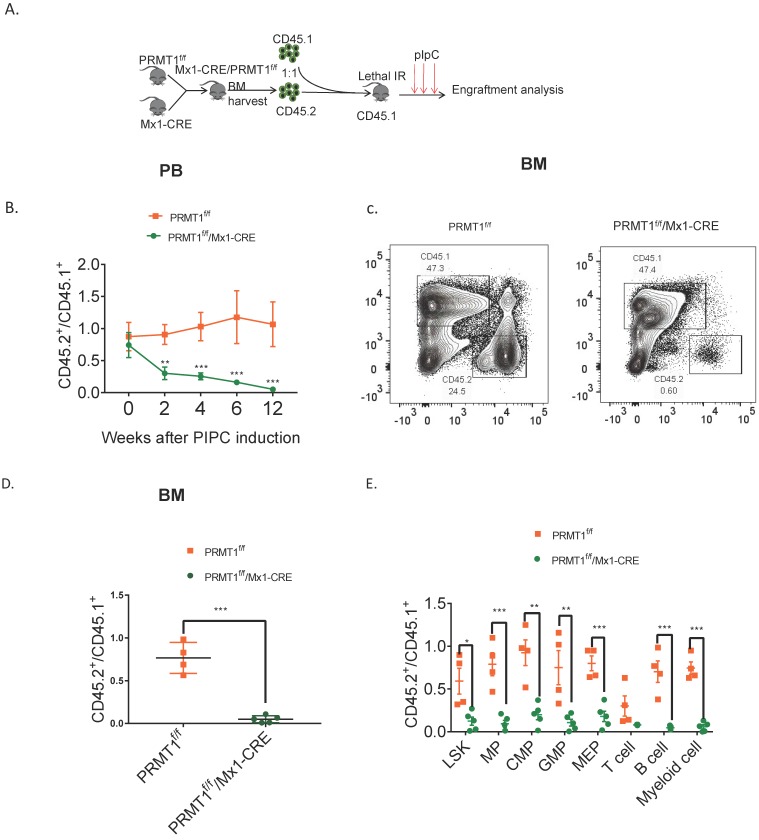
** Competitive repopulation assay to assess the hematopoietic consequences of PRMT1 deficiency *in vivo*.** (A) Scheme of the competitive repopulation assay. (B) Ratio of CD45.2/CD45.1 chimerism in the PB 2-12 weeks after competitive repopulation assay (*n*=5). **p<0.01, ***p<0.001. p values were determined by two-way ANOVA. (C) Representative FACS plots showing gating CD45.1 and CD45.2 population from PRMT1^f/f^/Mx1-CRE and of PRMT1^f/f^ mouse BM. (D) Ratio of CD45.2/CD45.1 chimerism in the BM of indicated recipient PRMT1^f/f^/Mx1-CRE (*n*=5) and PRMT1^f/f^ mice (*n*=4). ***p<0.001. p value was determined by Student's t test. (E) CD45.2 to CD45.1 ratio in LSK, MPs, CMP, GMP, MEP, T cell, B cell and myeloid cells of primary recipients at 12 weeks. Each dot represents an individual mouse transplanted with PRMT1^f/f^ (*n*=4) or PRMT1^f/f^/Mx1-CRE mouse (*n*=5) BM cells. *p<0.05, **p<0.01, ***p<0.001. p value were determined by Student's t test.

To further assess the long-term consequences of *Prmt1* deficiency in adult mouse hematopoiesis, we performed a competitive repopulation assay using unfractionated BM cells (Fig. 4A). We transplanted approximately 1 × 10^6^ BM cells isolated from Prmt1^f/f^, or PRMT1^f/f^/Mx1-CRE mice (which are CD45.2), together with equal numbers of WT CD45.1 competitor cells, into lethally irradiated CD45.1 recipient mice. Post pIpC induction, PRMT1-deleted CD45.2 cells were dramatically reduced compared to control donor cells, in both the peripheral blood and BM of recipient mice (Fig. 4B, C, D). Consistently, the chimerism contribution of PRMT1-deficient HSPCs or lineages was reduced relative to PRMT1-intact cells (Fig. 4E).

**Figure 5:**
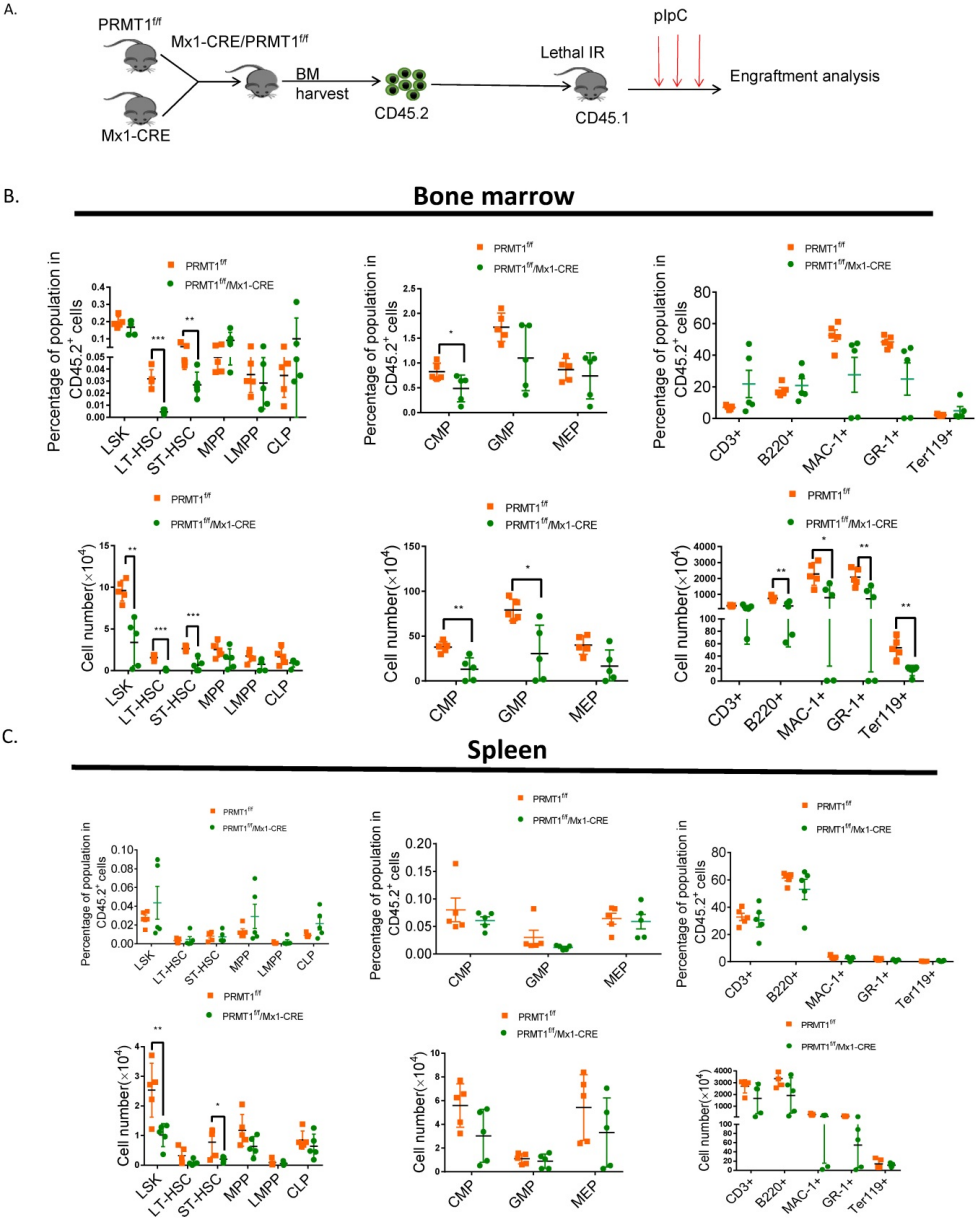
** Noncompetitive BM transplantation to assess the hematopoietic consequences of PRMT1 deficiency *in vivo*.** (A) Scheme of experimental design. (B) Percentage and number of CD45.2+ donor-derived population in the BM of PRMT1^f/f^/Mx1-CRE or PRMT1^f/f^ recipient mice 16 weeks after BM transplantation (*n*=5). *p<0.05, **p<0.01, ***p<0.001. p values were determined by Student's t test. (C) Percentage and number of CD45.2+ donor-derived population in the spleen of PRMT1^f/f^/Mx1-CRE or PRMT1^f/f^ recipient mice 16 weeks after BM transplantation (*n*=5). *p<0.05, **p<0.01. p values were determined by Student's t test.

To evaluate the long-term repopulation capacity of *Prmt1* null HSCs, we also performed noncompetitive BM transplantation. BM cells (2× 10^6^ cells) isolated from primary donor mice in which PRMT1 deletion was not induced, littermate controls were also included as a separate group. Total BM cells from PRMT1^f/f^/Mx1-CRE or control mice were harvested and transplanted into lethally irradiated CD45.1+ congenic recipients (Fig. 5A). Four weeks post BMT, PRMT1 deletion was induced by pIpC induction. Thereafter, we monitored donor cells in PB. Notably, CD45.2 cells were significantly reduced in PB of mice receiving PRMT1 KO BM cells (date not shown). We did CBC analysis at 4 weeks after pIpC injection, showing WBC was slightly decreased (Supplemental Fig. 3). While analyzing BM at 16 weeks post BMT, we found PRMT1 KO markedly reduced CD45.2+ donor chimerism of multiple lineages as well as HSPCs subsets relative to those of controls (Fig. 5B). In contrast, PRMT1 deletion had no marked effect on the frequency of CD45.2 cells in the spleen (Fig. 5C). In BM, the absolute numbers of HSPCs subsets and lineages were markedly reduced compared to littermate control mice (Fig. 5B). In spleen, the absolute numbers of LSK and ST-HSCs showed a significant decrease compared with similarly treated littermate control mice (Fig. 5C).

## Discussion

PRMT1 is known to be responsible for at least 85% of all arginine methylation catalyzation in mammalian cells 21. PRMT1 can regulate myeloid differentiation and lymphocyte development 22, 23, however, its function in HSC self-renewal as well as adult hematopoiesis has been unclear. In the present study, we generated a PRMT1 conditional KO mouse model. Using the Cre/lox-conditional system, we characterize the role of PRMT1 in regulating adult mouse HSC function. We found that the loss of PRMT1 expression leads to cytopenia and affects HSC repopulation and multilineage differentiation.

Our studies indicate that PRMT1 deficiency leads to a significantly impaired normal hematopoiesis. Adult HSCs are maintained in a quiescent state, which is important for their longevity and function 24. Doubling time analysis at homeostasis has shown that ST-HSCs and MPPs divide more frequently than LT-HSCs 25, 26. Moreover, our chimeric BM transplant experiments demonstrate that the PRMT1 deletion indeed decreased HSPCs *in vivo* repopulating capacity.

In addition, we found an erythroid maturation block in PRMT1^f/f^/ Mx1-CRE mice, in agreement with other's findings that, mediated through p38a, PRMT1 could promote lineage-specific differentiation toward erythrocytes 14, 15. Further supporting our results, others have demonstrated that inhibition of PRMT1 through RNAi depleted levels of the activating histone mark H4R3me and increased levels of the repressive H3K9me and H3K27me marks at the β-globin locus in erythrocytes^27^, contributing to an anemia phenotype. Regarding megakaryocyte maturation, we noticed that the frequency of MEPs was higher in PRMT1 KO compared with littermate controls. Additionally, through histology analysis of BM, we did find increased number of megakaryocytes. Our result is in agreement with others study, showing PRMT1 overexpression antagonized megakaryocytic differentiation 28. However, in our mouse studies, we found PRMT1 KO did not affect platelet level. Through mice femur histology analysis, we could not distinguish granular megakaryocyte or platelet-forming megakaryocyte. And the phenotypic marker of platelet-forming megakaryocyte is still not clear. Thus, we postulate that the platelet-forming megakaryocytes did not increase; conversely, the granular megakaryocyte may increase.

PRMT1 is an active component of signal transduction pathways including those from the B-cell receptor (BCR) 4, 18, 29-32. Our flow-cytometry analysis showed a significant decrease in the frequency and absolute number of mature B cells in PRMT1^f/f^/Mx1-CRE mouse BM, and, consistent with other's results 17, found that mice with a B-cell-specific deletion of PRMT1 display a partial block in B cell development at the pre-B cell stage, confirming that PRMT1 is required for lymphocyte development. Infantino et al 19 undertook an immunology analysis on mice with Prmt1 deletion in mature B cells. Their analysis revealed that PRMT1-catalyzed protein substrate methylation is essential for normal B cell proliferation, differentiation and survival. Taken together, our results show that PRMT1 is required for normal B cell development.

In the transplant context, PRMT1-deletion reduced HSPCs and lineage cell number evidenced by engraftment analysis (Fig. 5B), indicating that the hematopoietic changes in PRMT1 KO animals were caused by cell autonomous. Consistently, others also used transplant assay to evaluate the cell autonomous effect 33-35. But we can't exclude the possible non-cell autonomous effects.

Although knockout of *Prmt1* in mice can result in embryonic lethality at embryonic day 7.5^11^, ^21^, but *Prmt1*-conditional KO adult mice didn't die till pIpC induction 16 weeks (supplemental Fig. 2). These results suggested that PRMT1 inhibitors could be safely applied to hematological malignancies.

In conclusion, PRMT1 is required at multiple stages of hematopoietic differentiation. PRMT1 function is important for HSPCs' competitive fitness. Our results indeed identify that PRMT1 serves as a key epigenetic regulator of normal hematopoiesis that occurs throughout life.

## Supplementary Material

Supplementary figures.
