# Supplementary material for: Protein arginine methyltransferase 1 is required for maintenance of normal adult hematopoiesis
Source: Int J Biol Sci. 2019 Oct 23;15(13):2763–73. doi: 10.7150/ijbs.38859 (PMC6909962; doi:10.7150/ijbs.38859)

Figure S1.

Analysis of peripheral blood cell RBC and Hb counts at 0, 4, 6, 8, 10 and 12 weeks after pIpC injection are shown. *p<0.05, ***p<0.001. p values were determined by two-way ANOVA.

Figure S2.

Kaplan-Meier survival curve of PRMT1$^{f/f}$ (*n*=6), and PRMT1$^{f/f}$/Mx1-CRE mice (*n*=8).

Figure S3.

Analysis of peripheral blood cell WBC counts at 4 weeks after pIpC injection are shown (*n*=5).

# Supplementary figure S1

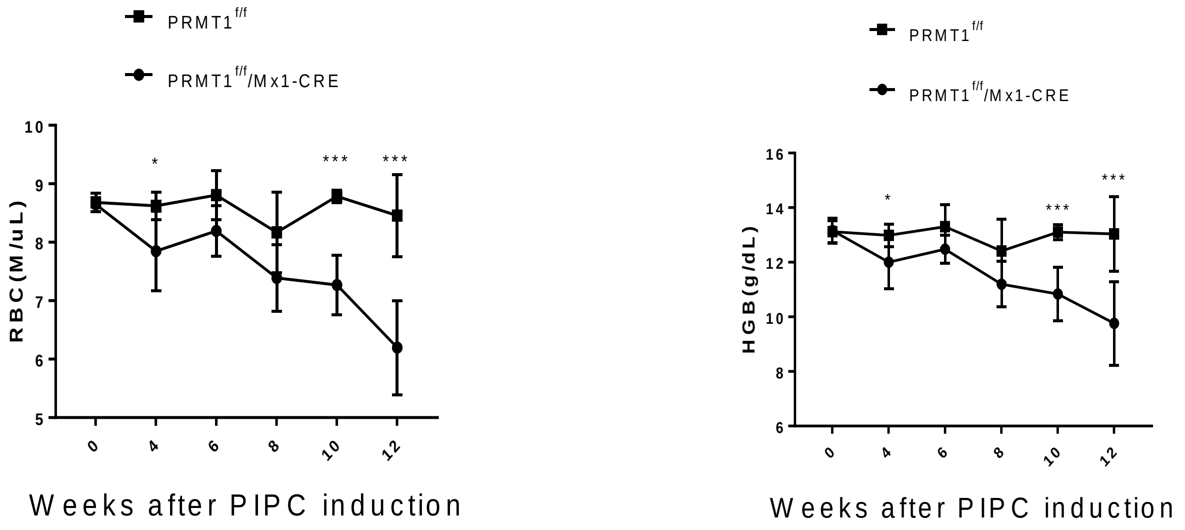

**Supplementary figure S2**

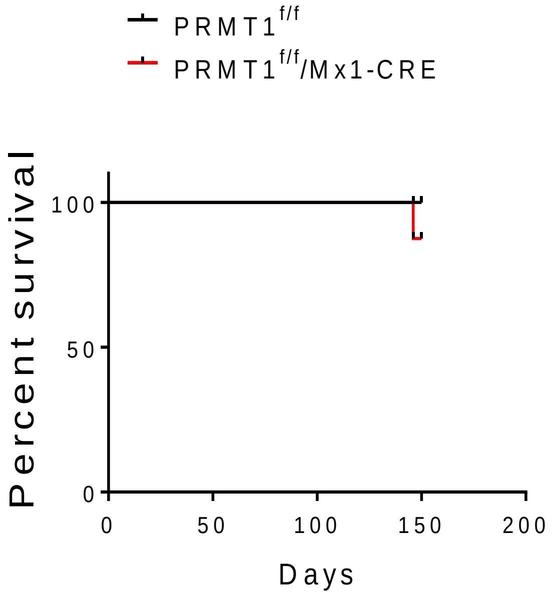

**Supplementary figure S3**

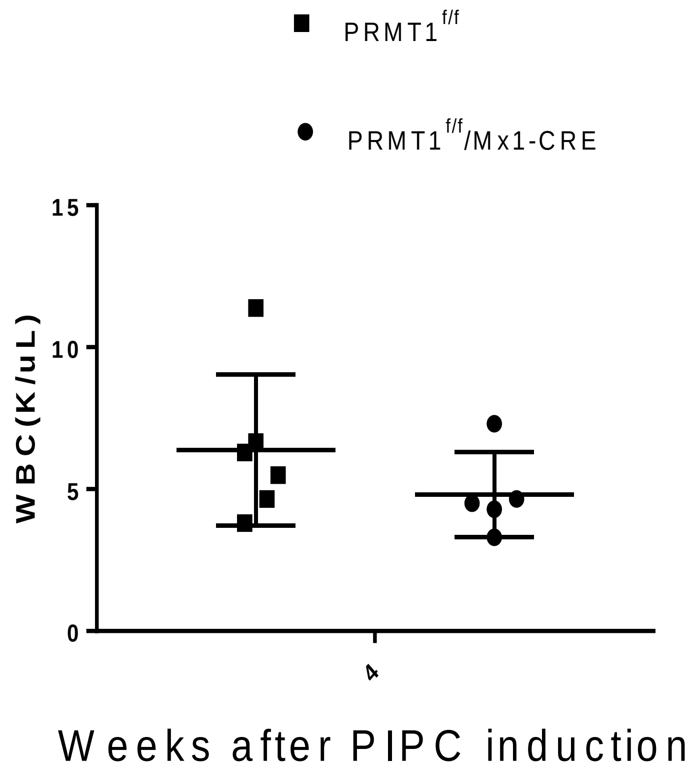

Supplement: Supplementary file 1 — Supplementary figures. [file ijbsv15p2763s1.pdf]
